# The Traditional Chinese Medicine Compound Huangqin Qingre Chubi Capsule Inhibits the Pathogenesis of Rheumatoid Arthritis Through the CUL4B/Wnt Pathway

**DOI:** 10.3389/fphar.2021.750233

**Published:** 2021-08-27

**Authors:** Xiao Wang, Jun Chang, Guoliang Zhou, Chenglong Cheng, Youyi Xiong, Jinfeng Dou, Gen Cheng, Chenggui Miao

**Affiliations:** ^1^ Department of Clinical Nursing, School of Nursing, Anhui University of Chinese Medicine, Hefei, China; ^2^ Department of Orthopaedics, 4th Affiliated Hospital, Anhui Medical University, Hefei, China; ^3^ Department of Pharmacy, School of Life and Health Sciences, Anhui University of Science and Technology, Fengyang, China; ^4^ Department of Pharmacology, School of Integrated Chinese and Western Medicine, Anhui University of Chinese Medicine, Hefei, China

**Keywords:** rheumatoid arthritis, cullin 4B, circ_0015756, canonical wnt signaling, huangqin qingre chubi capsule

## Abstract

The pathogenesis of rheumatoid arthritis (RA) is still not fully clarified, and the development of therapeutic drugs for RA is particularly urgent. Our group studies a possibility that circ_ 0015756/miR-942-5p may participate in the pathogenesis of RA through disordered *Cullin 4B* (*CUL4B*) and the traditional Chinese medicine compound Huangqin Qingre Chubi Capsule (HQC) may inhibit the pathogenesis of RA through the *CUL4B*/Wnt pathway. Data showed that the expression of circ_0015756 increased not only in fibroblast-like synoviocytes (FLS) of RA, but also in synovium and FLS of CIA mice, and the expression of miR-942-5p decreased. Abnormal circ_0015756 up-regulated the *CUL4B* expression and activated the canonical Wnt signaling pathway by inhibiting the expression of miR-942-5p. Circ_0015756 participated in the pathogenesis of RA and promoted the abnormal proliferation of FLS. Further, circ_0015756 activated the secretion of IL-1 and IL-8 and promoted the production of RA pathological gene *MMP3* and *fibronectin*. Further analysis showed that HQC inhibited the pathogenesis of RA through the *CUL4B*/Wnt pathway, and the specific target was *CUL4B*. HQC interfered with the effects of circ_0015756 on the pathogenesis of RA by inhibiting the *CUL4B*, showing a good therapeutic effect on RA.

## Introduction

Rheumatoid arthritis (RA) is a chronic and systemic autoimmune disease characterized by symmetrical and multi joint inflammation. The incidence rate of RA in the world is 1%, most of the patients are at 40–60 years old, which seriously threaten human health, especially the health of the elderly (Weyand and Goronzy., 2021). At present, the pathogenesis of RA is still not fully clarified. The existing research results of pathological mechanism have accelerated the research and development of therapeutic drugs. However, it is still urgent to investigate the pathogenesis of RA and develop new therapeutic drugs (Lin et al., 2020; Pandolfi et al., 2020).

Fibroblast-like synoviocytes (FLS) are the core effector cells in the pathogenesis of RA, and the abnormal proliferation of FLS is the initial factor of RA. FLS activation and proliferation play a key role in the pathological mechanism of RA. FLS with abnormal proliferation releases cytokines and chemokines such as IL-6, IL-8 and IL-15, and promotes the migration and activation of leukocytes from blood vessels to synovium (Guo et al., 2020b; Tang et al., 2020). FLS synthesizes and secretes extracellular matrix proteins such as fibronectin and cell adhesion molecule, and recruits and resides leukocytes to gather in joint synovium. FLS can activate B lymphocytes, secrete matrix metalloproteinase MMP-3 and matrix degrading enzyme, degrade articular cartilage and aggravate the condition of RA. FLS can enhance the immune response to the degradation of articular cartilage to produce specific antigens and promote vascular regeneration mediated by stromal cell-derived molecule (SDF-1) and fibroblast growth factor (de Oliveira et al., 2020; Pap et al., 2020).

Wnt signal plays an important role in regulating the pathological changes of RA. Wnts are glycoproteins that can bind to the cysteine rich region of the extracellular region of FZ protein on cell surface (Cici et al., 2019). Under the synergistic action of helper receptor LDL 5/6, Wnts initiate the gene transcription and up-regulate the cell proliferation through a series of intracellular signal transduction pathways. For example, abnormal expression of Wnt1 affects the pathological changes of RA. TCF reporter gene analysis shows that the Wnt1 protein induces the Wnt signal activation of FLS. The expression of *MMP3* is up-regulated by Wnt1 expression vector in normal human synovial cells, and the expression of *MMP3* is inhibited by adding anti-Wnt1 antibody or recombinant SFRP1 to synovial cell culture medium (Liu et al., 2021; Miao et al., 2013).

Wnt5a plays an important role in the pathological mechanisms of RA. Further, *in situ* hybridization and immunohistochemical analysis show that wnt7b is highly expressed in RA articular cartilage, bone and synovium. After wnt7b is transfected into RA FLS, the TNF-α, IL-1 β and IL-6 are significantly up-regulated, suggesting that wnt7b plays an important regulatory role in the pathogenesis of RA (Ma et al., 2018; Marquez and Chen, 2020).

Among the eight cullins (cul1-7 and PARC) in higher organisms, the CUL4 subfamily of cullin-really interesting new gene (RING) ubiquitin ligase (CRL) contains two family members, *CUL4A* and *CUL4B*, which have extensive sequence homology. In recent years, a large number of studies have confirmed that CUL4B is closely related not only to cancer pathology, but also to the pathogenesis of RA (Lin et al., 2019).

Huangqin Qingre Chubi Capsule (HQC) is a traditional Chinese medicine compound for the treatment of RA created by the First Affiliated Hospital of Anhui University of Chinese medicine. HQC is composed of five traditional Chinese medicines: *Scutellaria baicalensis Georgi*, *Gardenia jasminoides Ellis*, *Coicis Semen*, *Semen Persicae* and *Clematis chinensis Osbeck* (Guo et al., 2020a). Clinical practice has proved that HQC can reduce the pain index, reduce the joint swelling, reduce the shorten morning stiffness time and improve the joint function in RA patients. HQC can also improve the lung function, heart function, and the anemia, inhibit the platelet activation, improve the hypercoagulable state and lipoprotein metabolism (Huang et al., 2020).

When screening the targets of HQC, our research group found that abnormally low expression of *β-catenin*, a key gene of Wnt signaling pathway, was detected in the synovium of CIA mice after HQC gavage. Meanwhile, HQC gavage of CIA mice also inhibited the expression of Wnt signal pathway genes *c-Myc* and *CCND1*. HQC gavage of CIA mice also decreased the expression of CUL4B in mouse synovium. Previous studies in our group confirmed that the abnormal CUL4B had an effect on the canonical Wnt signaling pathway.

Therefore, we propose a hypothesis that HQC may inhibit the pathogenesis of RA through the *CUL4B*/Wnt pathway, and HQC may be an effective RA therapeutic drug dependent on the *CUL4B*/Wnt pathway. In addition, we also deeply studied the regulatory mechanisms before the *CUL4B*/Wnt pathway. This study is of significance to clarify the pathogenesis of RA, and also provides a basis for HQC as an effective compound drug for the treatment of RA.

## Materials and Methods

### Materials and Reagents

Anti-CUL4B antibody (ab227724), Anti-beta Catenin antibody (ab32572), Anti-c-Myc antibody (ab32072), Anti-Cyclin D1 antibody (ab16663), Anti-MMP3 antibody (ab52915), Anti-Fibronectin antibody (ab2413) and Anti-beta Actin antibody (ab8226) were purchased from Abcam (Waltham, MA, United States). miR-942-5p mimics, inhibitors and negative control (miRNA NC) were purchased from Shanghai GenaPharma Pharmaceutical Technology Co., Ltd. (Shanghai, China). *CUL4B, β-catenin, MMP3, FIBRONECTIN*, *c-Myc, CCND1 and β-ACTIN* primers were produced by Shanghai Sangon Biological and Technological Company (Shanghai, China). MTT [3-(4,5-dimethylthiazol-2-yl)-2,5- diphenyltetrazoliumbromide] and DMSO (dimethyl sulfoxide) were purchased from Sigma Inc.(St. Louis, MO, United States).

### Preparation of Collagen-Induced Arthritis Model and Fibroblast-Like Synoviocytes Culture

5 mg/ml Freund’s complete adjuvant and 2 mg/ml chicken type II collagen were mixed in equal volume. After equal volume mixing, the emulsion was prepared by high-speed agitator. SPF grade C57BL male mice were purchased, and the 0.1 ml emulsion was injected into the tail root of the mice for immunization. After 21 days, another 0.1 ml was injected again to strengthen the immunization. After 35 days, CIA model mice were successfully prepared (Miyoshi and Liu, 2018).

Then the model mice in the experimental group were killed, the synovial tissue was isolated, and FLS was cultured by tissue block method. FLS was cultured in a cell culture flask with 5% CO_2_ at 37°C in a high sugar DMEM medium supplemented with 15% (V/V) heat inactivated fetal bovine serum (FBS) and penicillin streptomycin solution. The experimental cells we used were the third to sixth generations of FLS. The animal experiment of this study was carried out by an agreement approved by the Animal Ethics Committee of Anhui University of Chinese medicine.

### Huangqin Qingre Chubi Capsule Administration Method and Preparation of Huangqin Qingre Chubi Capsule Medicated Serum

Based on the clinical dosage of HQC in RA patients and the body surface area exchange algorithm of human and mice, the dosage of CIA mice was determined as 0.36 g/kg weight per day. Then forty SPF grade C57BL male mice were randomly divided into two groups: blank control group and HQC group, with 20 mice in each group. HQC (0.36 g/kg weight) was administered by gavage (gavage volume: 10 ml/kg). The daily dose was administered by gavage twice, and the control group was given normal saline. Each group received continuous administration for 3 days, and then blood was taken from the femoral artery after anesthesia. Centrifugation, filtration, 56°C water bath for 30 min to inactivate complement. Mix well in the same group, 0.22 μM filter membrane for sterilization and seal at −80°C for storage. The dose of HQC medicated serum added to FLS culture medium is 20% of the final concentration of culture medium. The control group was added with the same volume of cell culture medium without drug containing serum.

### Transient Transfection of miR-942-5p Mimics and Inhibitors

MiR-942-5p mimics, inhibitors and negative control sequence (miRNA NC) were purchased from Shanghai Gena Pharmaceutical Technology Co., Ltd. (Shanghai, China). These miRNAs were transfected into cultured FLS through Lipofectamine™ 3,000 (Invitrogen, Carlsbad, CA, United States) in accordance with the manufacturer’s instructions. FLS were transfected with a density of 0.5-1 × 10^5^ cells/mL in DMEM containing 15% FBS. After 6 h of transfection, the culture medium was discarded, and then the transfected FLS was cultured for the specified time for further analysis.

### MTT Assay

Each group of treated FLS with a cell density of about 0.5-1 × 10^5^/ml was cultured in 96 well plates for 24 h. Then FLS and 20 μL MTT (5 mg/ml) were cultured in cell incubator for another 4 h. After incubation, FLS containing MTT reagent was resuspended at 150 μL DMSO (Sigma, United States) and shaked the bed at low speed for 10 min to completely dissolve the MTT transformed formazan. The absorbance was measured at 490 nm using a Thermomax microplate reader (bio-tek EL, United States), and the detected value indirectly reflected the number of living cells.

### Real Time Quantitative PCR

We prepared total RNA using Trizol reagent (Invitrogen, United States) according to the manufacturer’s instructions, and reverse transcribed the total RNA according to the instructions provided by revertaid first strand cDNA synthesis kit (Fermentas, United States). Then, quantifast ^®^ SYBR ^®^ The green PCR kit and the following primers were used for real-time qPCR analysis of the target gene:

*CUL4B* forward: 5′-GCC​CCT​GGA​ATA​GAG​GAT​GGA-3′, reverse: 5′-TCG​GTC​TGT​AGT​GCT​TGC​TTG​T-3′;
*β-catenin* forward: 5′-CTTAC GGCAATCAGGAAAGC-3′, reverse: 5′-ACAGAC AGCACCTTCAGCACT-3’;
*MMP3* forward: 5′-TGAT GAACG ATGGACAGATGA-3′, reverse: 5′-AGC​ATT​GGC​TGA​GTG​AAA​GAG-3’;
*fibronectin* forward: 5′-GACACTATGCGGGTCA CTTG-3′, reverse: 5′-CCCAGG CAGGAGATTTGTTA-3’;
*c-Myc* forward: 5′-ATT​TCT​ATC​ACC​AGC​AAC​AGC​A -3′, reverse: 5′-ATT​TCT​ATC​ACC​AGC​AAC​AGC​A-3’;
*CCND1* forward: 5′-GCC​CTC​CGT​TTC​TTA​CTT​CAA-3′, reverse: 5′-CTCTTCGC ACTTCT GCTCCTC-3’;
*β-ACTIN* forward: 5′-CCC​ATC​TAT​GAG​GGT​TAC​G C-3′, reverse: 5′-TTT​AAT​GTC​ACG​CAC​GAT​TTC-3’;
circ_0015756 forward: 5′-TGG​ACG​GAA​CCA​CCT​CAA​TG-3′, reverse: circ_0015756-R: 5′-CCT​GAA​ACC​ACC​CTC​ACA​AGT-3′;
miR-942-5p forward: 5′-AGG​GTC​TTC​TCT​GTT​TTG​GC-3′, reverse: 5′-GTT​GTG​GTT​GGT​TGG​TTT​GT-3′;
*U6* forward:5′-CTCGCTTCGGCAGCACA-3′, reverse: 5′-AAC​GCT​TCA​CGA​ATT​TGC​GT-3′.

Subsequently, the expression level was calculated using the 2^−ΔΔCt^ method. The amplification steps were based on the method provided by the kit, and the amplifcation procedure include 95°C for 10 min, followed by 40 cycles at 95°C for 15 s, 60°C for 30 s, 72°C for 30 s. In particular, the PCR amplifcation procedure for circ_0015756 was 95°C for 10 min, followed by 40 cycles of 95°C for 10 s, 60°C for 15 s, and 72°C for 10 s. The results were repeated three times independently.

### Western Blot Analysis

Referring to the method provided by the cell lysis kit (Beyotime, China), the protein samples of each group to be analyzed are lysed, and the protein concentration was determined using the enhanced BCA protein analysis kit (Beyotime, China). Then, the total proteins of each group were separated by SDS-PAGE and imprinted on PVDF membrane for western blot analysis. Nitrocellulose blots were incubated in the corresponding antibody dilution buffer for 6 h, including CUL4B, β-catenin, c-Myc, cyclin D1, MMP3 and fibronectin, and these corresponding antibodies were diluted from 1:500 to 1:800. Mouse monoclonal antibodie β-actin was used at a dilution ratio of 1:800. Western blot was detected by ECL chemiluminescence kit (ECL-plus, Thermo Scientific), and the gray value of each band was further analyzed.

### Enzyme-Linked Immuno Sorbent Assay

ELISA was used to determine the levels of IL-1, IL-6 and IL-8 produced in RA FLS or CIA FLS. The quantitative ELISA kit was purchased from Shanghai Yuanye Biotechnology Co., Ltd. (Shanghai, China) the detection procedure referred to the method provided in the kit manual. The optical density detected by the detector was 450 nm, and three repeated measurements were made for each experimental group.

### Construction and Transfection of *CUL4B* Vectors

CUL4B sequences were amplified by PCR, and then the PCR products were subcloned into pEGFP-C2 vectors for overexpression analysis according to standard instructions. The constructed pEGFP-C2 vectors containing the *CUL4B* mutation sequence were synthesized by the QuikChange Site-Directed Mutagenesis Kit (Stratagene, United States). Then the constructed expression vectors or mutant vectors were transfected into RA FLS or CIA FLS for further gene regulation analysis. All primers and pEGFP-C2 vectors were purchased from Shanghai gene Pharmaceutical Co., Ltd. (Shanghai, China), and all constructs were confirmed by sequencing.

### Dual-Luciferase Reporter Assay

The wild-type luciferase reporter genes (circ_0015756-WT and *CUL4B* 3′UTR-WT) were formed by cloning circ_0015756 or CUL4B 3′UTR containing miR-942-5p binding site into pmirGLO vectors (LMAIBio, Shanghai, China). The mutant luciferase reporters (circ_0015756-MUT and *CUL4B* 3′UTR-MUT) were prepared via corresponding mutated binding sites. Subsequently, the corresponding reporter gene and miRNA NC or miR-942-5p were co-transfected into RA FLS. Firefy and Renilla luciferase activities were determined by Dual-Lucy Assay Kit (Solarbio, Beijing, China). Firefy luciferase activity was standardized by Renilla luciferase activity.

### Ribo Nucleic Acid Immunoprecipitation Assay

EZ-Magna RIP kit (Millipore, MA, United States) is used for RIP analysis, and the method refers to the instructions provided by the manufacturer. After lysis of RA FLS in RIP lysis bufer, cell lysates were treated with magnetic beads combined with Ago2 antibody (anti-Ago2) overnight at 4°C. The corresponding antibody for negative test is IgG antibody (anti-IgG). The precipitated RNA was purified and the content of the target product was analyzed by RT-qPCR.

### Ribo Nucleic Acid Pull-Down Assay

The binding of miR-942-5p to circ_0015756 was analyzed by RNA pull-down assay. Biotinylated miR-942-5p (bio-miR-942-5p) and negative control miRNA (bio-miRNA NC) were purchased from GenePharma (Shanghai, China). RA FLS was lysed and incubated with streptavidin coated magnetic beads (Invitrogen, CA, United States). Then, the abundance of circ_0015756 was measured by RT-qPCR analysis.

### Statistical Analysis

Statistical significance was determined by either the Student’s t-test for comparison between means or one-way analysis of variance with a post hoc Dunnett’s test. Data are represented as Mean ± SE. Significance was defined as *p* < 0.05 vs. controls.

## Results

### Huangqin Qingre Chubi Capsule Ameliorates the Severity of Arthritis of CIA Mice

The effects of HQC on CIA mice were evaluated, the evaluation indexes included the incidence rate, arthritis score, hind foot swelling, paw withdrawal threshold and body weight.

**FIGURE 1:**
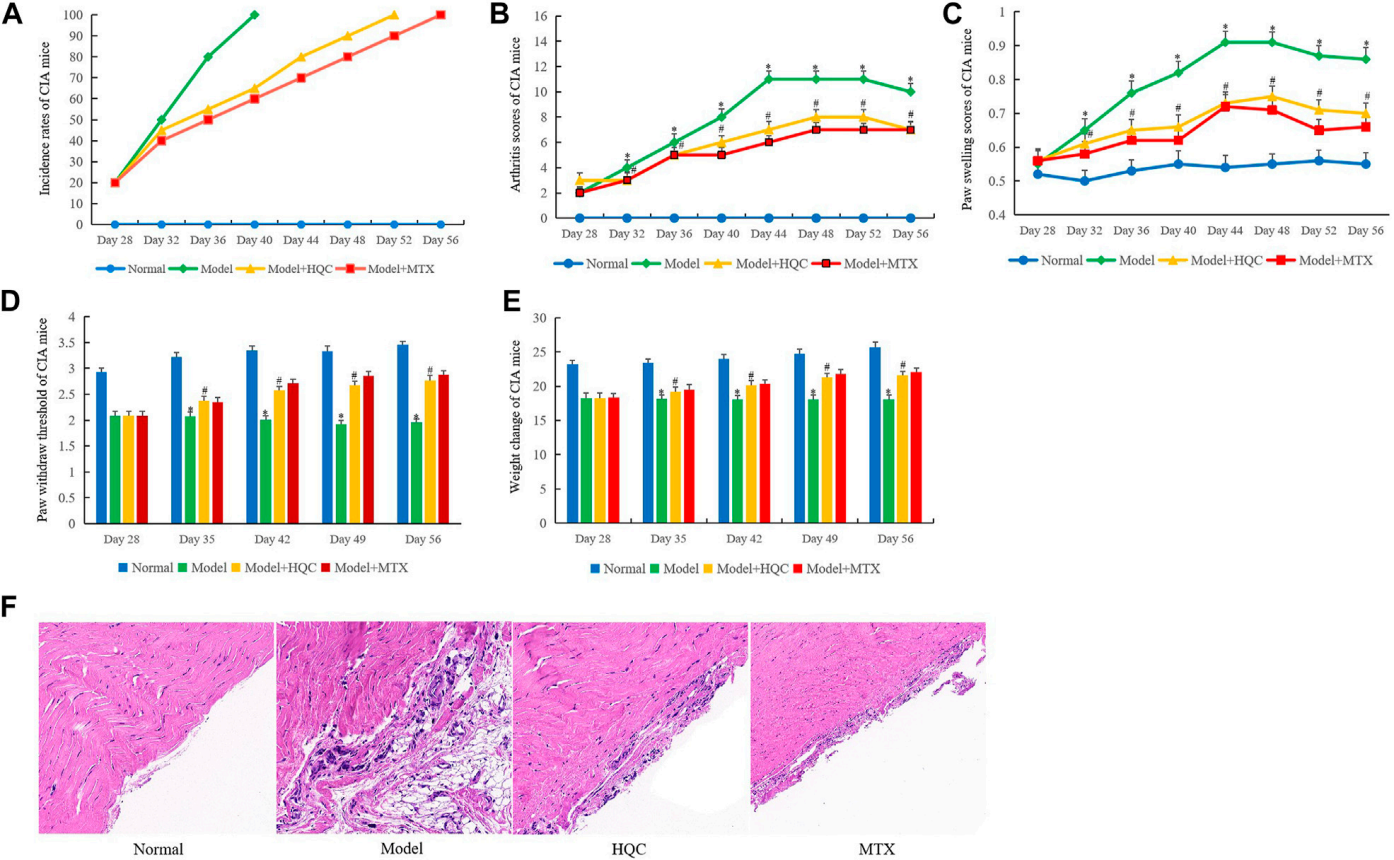
HQC ameliorates the severity of arthritis of CIA mice. The effects of HQC on CIA mice were evaluated, including the incidence rate, arthritis score, paw swelling score, pain sensitivity and body weight. **(A)**, the effects of HQC on the incidence rates of CIA mice were evaluated; **(B)**, arthritis score of mice in each group; **(C)**, paw swelling score in each group; **(D)**: the pain sensitivity of each group was characterized by paw withdraw threshold; **(E)**: the health status of each group was evaluated by the weight change of mice; **(F)**: the effects of HQC on synovial pathology of model mice was evaluated by HE staining, “Normal” is normal group, “Model” is model group, “HQC” is HQC treatment group, “MTX” is positive drug MTX group. *Model group was compared with normal group; # HQC group was compared with model group.

MTX has a significant effect in the treatment of RA, can alleviate the symptoms of RA and reduce joint injury. Therefore, MTX was used as a positive control drug in this study (Friedman and Cronstein, 2019). As shown in Figure 1A, the incidence rate of CIA mice in model group reached 100% on the 40th day. The incidence rate of HQC treatment group reached 100% on the 52nd day and methotrexate (MTX) group was the 56th days. The overall evaluation showed that HQC could delay the onset of CIA in mice. In the arthritis score (Figure 1B) and paw swelling score (Figure 1C), from the 32nd day, the score of the model group was significantly higher than that of the normal group, and the scores of arthritis and paw swelling in the HQC group and MTX group were significantly lower than those in the model group. The pain sensitivity of each group of CIA mice was characterized by paw withdraw threshold (Figure 1D), and the health status of each group was evaluated by the weight change of mice (Figure 1E). The data showed that from the 35th day, the two indexes of the model group were significantly lower than those of the normal group, and the indexes of the HQC group and MTX group were significantly higher than those of the model group.

HE staining showed that compared with the normal group, the synovium of CIA mice in the model group was seriously damaged, synovium proliferation was obvious, FLS arrangement was disordered and loose, and a large number of inflammatory cells were infiltrated. After HQC treatment, the severity of synovial damage, synovial hyperplasia, FLS proliferation and inflammatory cell infiltration in CIA mice were significantly reduced. As a positive control drug, MTX may be more effective than HQC (Figure 1F). However, HQC still has considerable therapeutic effect on CIA mice.

### Huangqin Qingre Chubi Capsule Inhibited the Proliferation of Rheumatoid Arthritis Fibroblast-Like Synoviocytes

**FIGURE 2:**
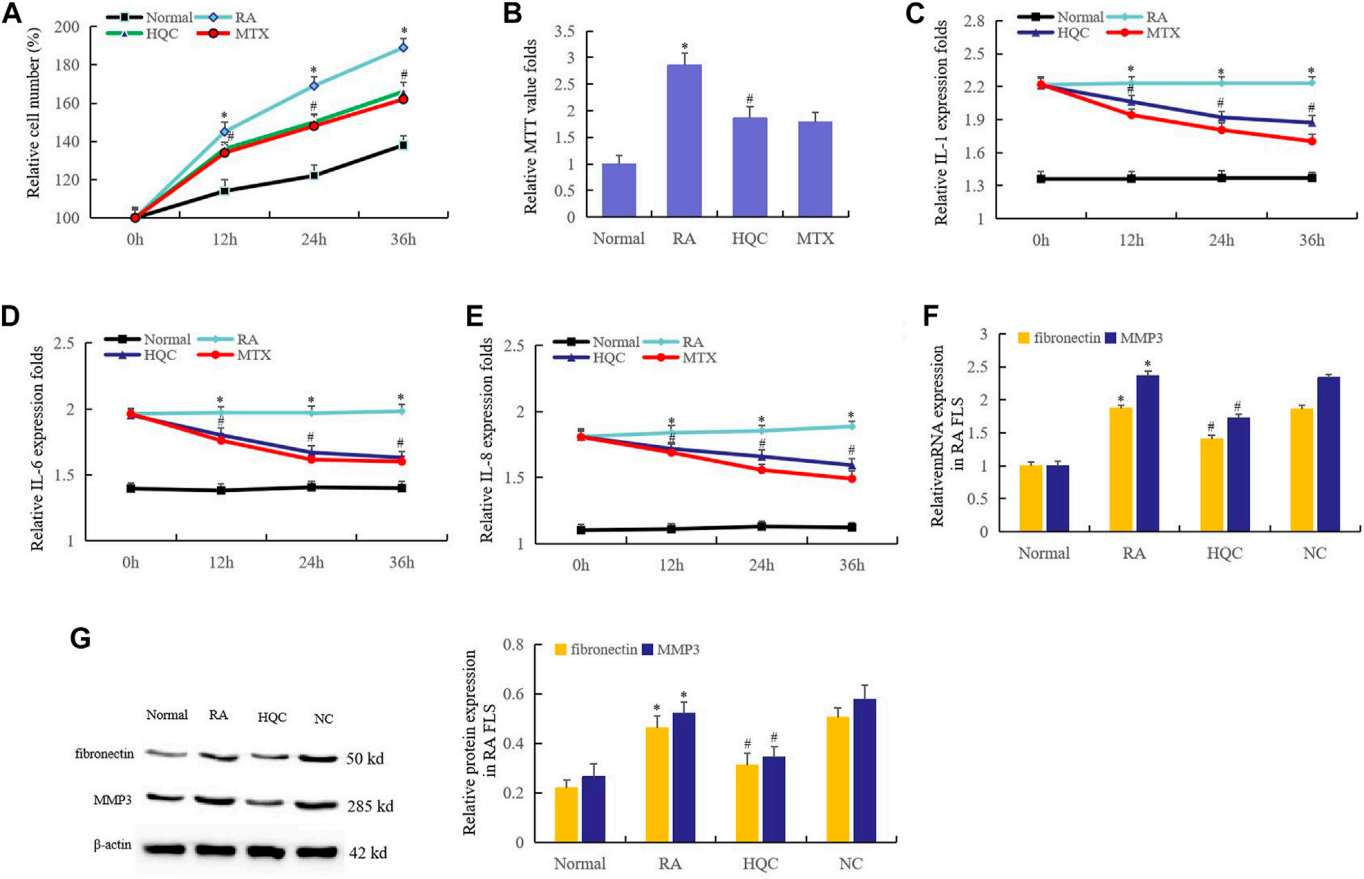
HQC inhibited the proliferation of RA FLS. The effects of HQC on RA was evaluated using RA FLS. **(A)**: the effect of HQC on RA FLS growth was detected by cell counting; **(B)**: the effect of HQC on RA FLS growth was detected by MTT; **(C)**: the effect of HQC on the IL-1 secretion was detected by ELISA; **(D)**: ELISA was used to detect the effect of HQC on IL-6 secretion; **(E)**: the effect of HQC on the IL-8 was detected by ELISA; **(F)**: the roles of HQC in the expression of *MMP3* and *fibronectin* at the RNA levels were detected by real time qPCR; **(G)**: the roles of HQC in the expression of *MMP3* and *fibronectin* at the protein levels were detected by western blotting. *RA FLS group was compared with normal group; # HQC group was compared with RA FLS group.

The therapeutic effects of HQC on CIA mice suggest that HQC may inhibit the pathological development of RA. Therefore, we first investigated the effects of HQC on RA FLS growth by cell counting. The results showed that compared with normal FLS, the proliferation of RA FLS increased significantly. In RA FLS, the proliferation of FLS in HQC group and MTX group were lower than that in RA group (Figure 2A). These findings were further confirmed by MTT analysis (Figure 2B). The effects of HQC on the production of IL-1, IL-6 and IL-8 in RA FLS were determined by ELISA. The levels of IL-1 (Figure 2C), IL-6 (Figure 2D) and IL-8 (Figure 2E) in RA FLS treated with HQC for 12, 24 and 36 h were significantly down-regulated. *MMP3* and *fibronectin* are pathological related genes in RA, and overexpression of these two genes indicates the development of RA pathology. We found that HQC significantly inhibited the expression of *MMP3* and *fibronectin* at the RNA (Figure 2F) and protein (Figure 2G) levels in RA FLS. These results suggest that HQC has therapeutic effect on RA and may be a potential natural product for the treatment of RA.

### Huangqin Qingre Chubi Capsule Inhibited the *CUL4B* Expression in CIA Mice and Rheumatoid Arthritis Fibroblast-Like Synoviocytes

**FIGURE 3:**
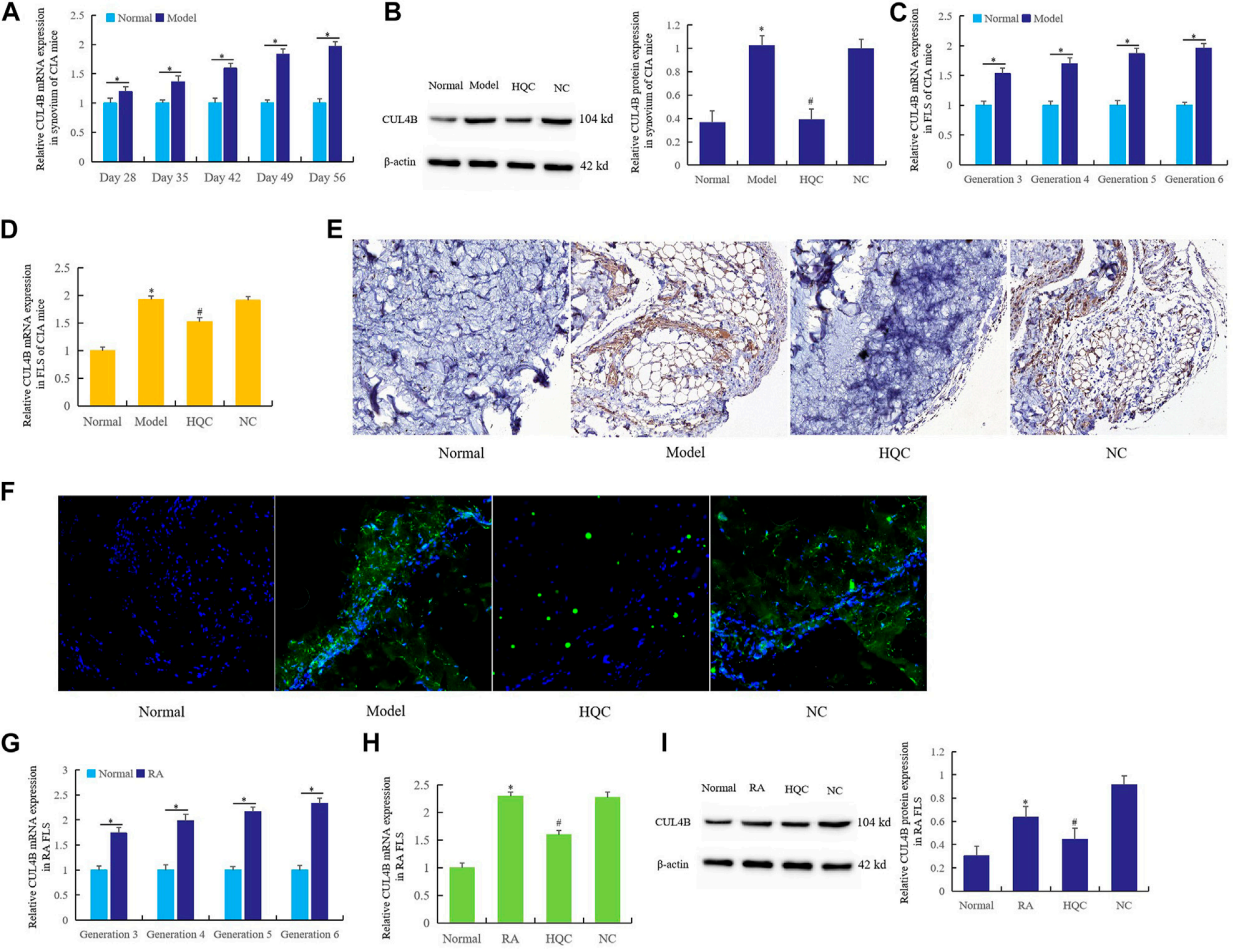
HQC inhibited the *CUL4B* expression in CIA mice and RA FLS. The effects of HQC on *CUL4B* expression was evaluated using samples from CIA mice and RA FLS. **(A)**: Ten CIA mice were killed every 7 days from the 28th day to the 56th day, then the expression of *CUL4B* in synovium was detected by real time qPCR; **(B)**: the expression of *CUL4B* in synovium of CIA mice in each group was detected by western blotting; **(C)**: the expression of *CUL4B* in the third to sixth generation CIA FLS was detected by real-time qPCR; **(D)**: the effect of HQC on *CUL4B* expression was detected by real-time qPCR; **(E)**: the role of HQC in the expression of *CUL4B* was detected by immunohistochemistry; **(F)**: immunofluorescence was used to detected the role of HQC in the expression of *CUL4B*, “Normal” is normal group, “Model” is model group, “HQC” is HQC treatment group, “NC” is the pure water group without HQC; **(G)**: Real time qPCR was used to detect the expression of *CUL4B* in RA FLS; real-time qPCR **(H)** and western blotting **(I)** were used to detected the effects of HQC on the expression of *CUL4B* in RA FLS. For **(B,C,D,H,I)**, *Model/RA FLS group was compared with normal group; # HQC group was compared with model/RA FLS group.

The changes of *CUL4B* expression and the effects of HQC on *CUL4B* expression were first detected in the synovium and FLS of CIA mice. Ten CIA mice were killed every 7 days from the 28th day to the 56th day, and the expression of *CUL4B* in synovium was detected by real time qPCR (Figure 3A). Another group of mice were given HQC from the 28th day to the 56th day, then the expression of *CUL4B* in synovium of mice in each group was detected by western blotting (Figure 3B). The results showed that the expression of *CUL4B* in synovium of CIA mice increased gradually with the prolongation of modeling time. Intragastric administration of HQC could significantly inhibit the expression of *CUL4B* in synovium of CIA mice.

Real time qPCR was used to detect the expression of *CUL4B* (Figure 3C) in the third to sixth generation CIA FLS. HQC medicated serum (20%) was added to FLS culture medium for the fourth generation. After 36 h of culture, the effect of HQC on the *CUL4B* expression was detected by real-time qPCR (Figure 3D). The results showed that the expression of *CUL4B* gradually increased from the third to the sixth generation FLS, and HQC significantly inhibited the expression of *CUL4B* in the pathogenesis of CIA mice.

The results of immunohistochemistry (Figure 3E) and immunofluorescence (Figure 3F) showed that compared with the normal group, the expression of *CUL4B* in the synovium of CIA model mice was significantly increased, and the expression of *CUL4B* was significantly decreased after intragastric administration of HQC.

Real time qPCR was used to detect the expression of *CUL4B* in RA FLS from the third to sixth generation (Figure 3G). HQC medicated serum (20%) was added to the culture medium of RA FLS of the fourth generation. After 36 h of culture, the expression of *CUL4B* was detected by real-time qPCR (Figure 3H) and western blotting (Figure 3I). The expression of *CUL4B* gradually increased in RA FLS from the third and sixth generation, and the HQC significantly inhibited the expression of *CUL4B* in RA FLS. These findings suggest that HQC may affect the pathological development of RA by regulating *CUL4B* expression.

### Huangqin Qingre Chubi Capsule Inhibited the Wnt Signaling Pathway in CIA Mice and Rheumatoid Arthritis Fibroblast-Like Synoviocytes

**FIGURE 4:**
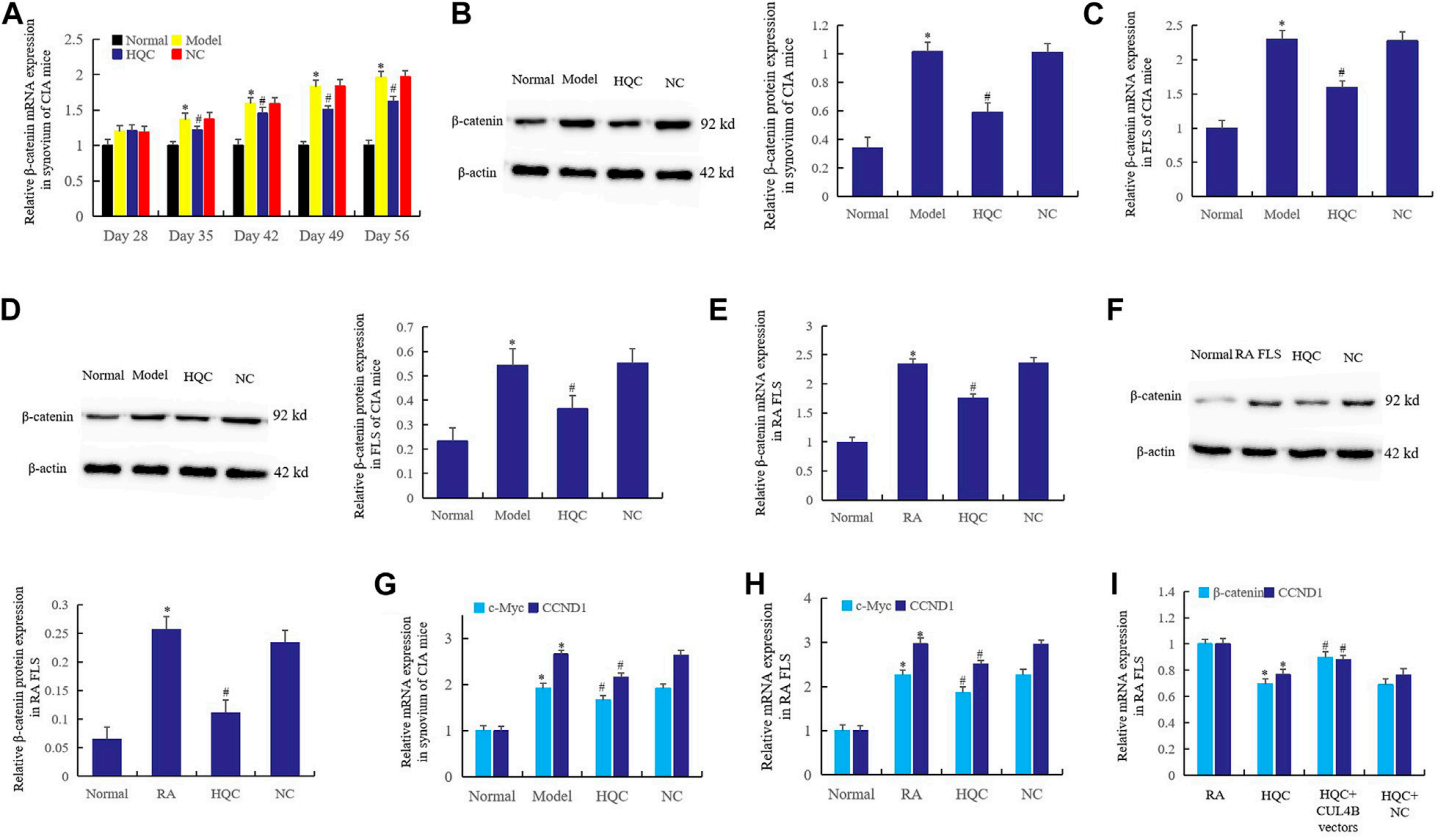
HQC inhibited the Wnt signaling pathway in RA pathogenesis. The effect of HQC on the expression of *β-catenin* was detected in synovium from CIA model mice by real-time qPCR **(A)** and western blotting **(B)**; Real time qPCR **(C)** and western blotting **(D)** were used to detected the effect of HQC on the expression of *β-catenin* in CIA FLS; the effect of HQC on the *β-catenin* was detected in RA FLS by real-time qPCR **(E)** and western blotting **(F)**; **(G)**: the expression of *c-Myc* and *CCND1* in the synovium of CIA mice was detected by real-time qPCR; the expression of *c-Myc* and *CCND1* in RA FLS was detected by real-time qPCR **(H)**; the interference of *CUL4B* vectors to HQC was detected by real-time qPCR **(I)**. *Model/RA FLS group was compared with normal group; # HQC group was compared with model/RA FLS group. For **(I)**, *HQC group was compared with RA FLS group; # HQC + *CUL4B* vector group was compared with HQC group.

HQC was administered by gavage from the 28th day of CIA model preparation, and the expression of *β-catenin* was detected on the 28th, 35th, 42nd, 49th and 56th days respectively. The results showed that HQC inhibited the *β-catenin* expression in synovium of CIA model mice from the 35th day (Figure 4A). Western blotting detection in each group showed that HQC could significantly reduce the *β-catenin* expression on the 56th day of treatment (Figure 4B). Furthermore, the results of real time qPCR (Figure 4C) and western blotting (Figure 4D) showed that HQC medicated serum (20%) added to CIA FLS culture medium of each group significantly inhibited the expression of *β-catenin*. Adding HQC medicated serum (20%) to the cell culture medium of cultured RA FLS can also inhibit the expression of *β-catenin* (Figures 4E,F).

In addition, the expression of *c-Myc* and *CCND1* in the synovium of CIA mice decreased significantly after intragastric administration of HQC (Figure 4G). The same phenomenon was observed when HQC medicated serum (20%) was added to RA FLS medium (Figure 4H). This suggests that HQC inhibits the canonical Wnt signaling pathway in the pathogenesis of RA. When HQC medicated serum (20%) was added to cultured RA FLS or *CUL4B* vectors were transfected after HQC medicated serum (20%) was added, it was found that HQC medicated serum (20%) could inhibit the expression of *β-catenin* and *CCND1*, but *CUL4B* vectors reversed the effects of HQC medicated serum (20%) (Figure 4I). This suggests that *CUL4B* plays a key role in the mechanisms of HQC in the treatment of RA.

### *CUL4B* is a Target of miR-942-5p in Rheumatoid Arthritis Fibroblast-Like Synoviocytes

**FIGURE 5:**
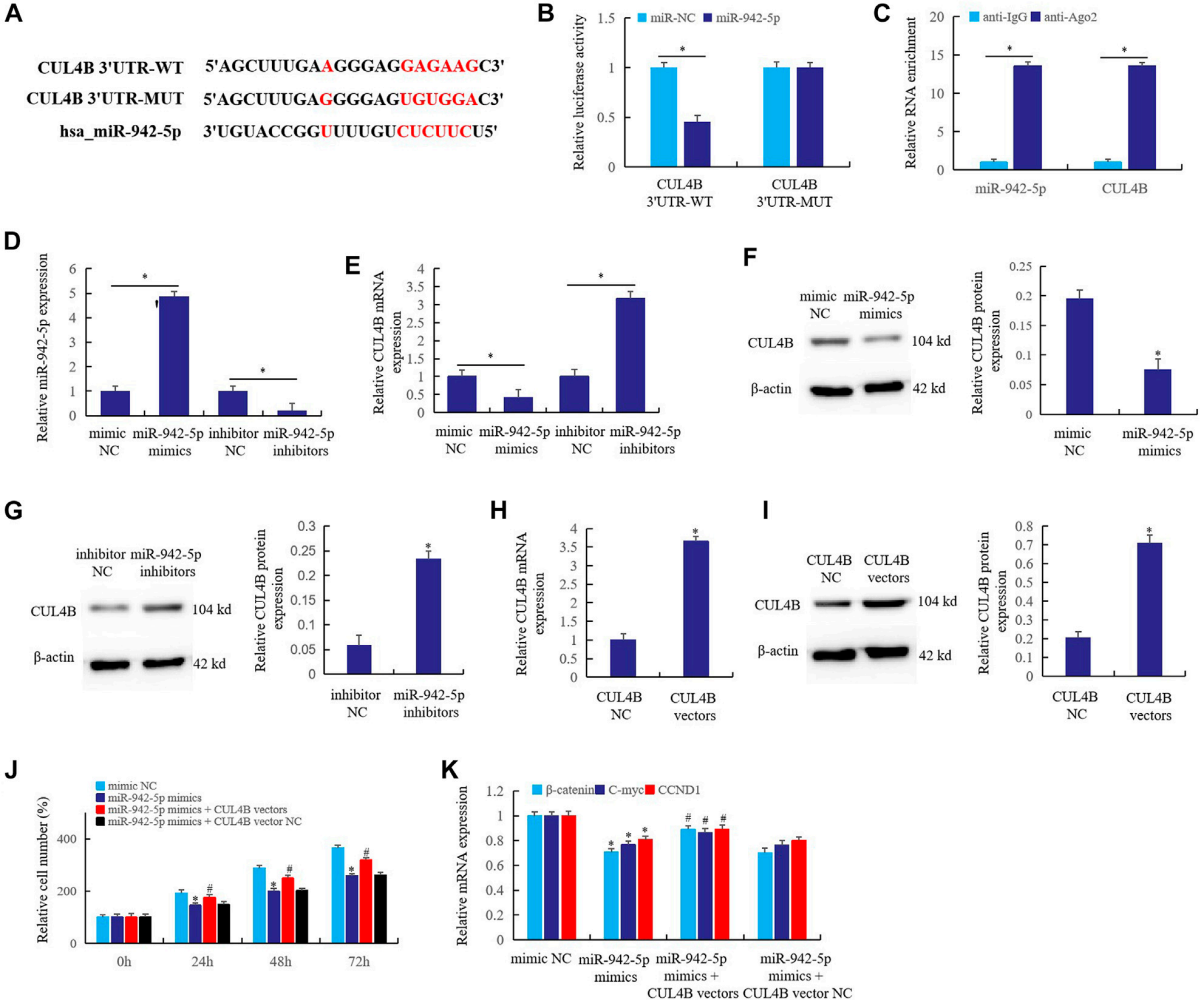
*CUL4B* is a target of miR-942-5p in RA FLS. **(A)** the interaction relationship of the *CUL4B* and miR-942-5p was predicted by bioinformatics; **(B)**: double luciferase reporter gene; **(C)**: RIP assay; the effects of mir-942-5p mimics or inhibitors on the miR-942-5p **(D)** or CUL4B **(E)** was detected by real-time qPCR; **(F,G)**: western blotting; **(H,I)**: the roles of *CUL4B* vectors in the *CUL4B* expression were detected; **(J,K)**: the reversal effects of *CUL4B* vectors on miR-942-5p mimics were detected by cell counting and real-time qPCR. For **(J,K)**, *miR-942-5p mimics group was compared with mimic NC group; # miR-942-5p mimics + *CUL4B* vector group was compared with miR-942-5p mimics group.

Bioinformatics predicts that *CUL4B* may be a direct target of miR-942-5p (Figure 5A). To verify this prediction, double luciferase reporter gene (Figure 5B) and RIP assay (Figure 5C) were used to confirm the direct regulatory relationship between miR-942-5p and *CUL4B*. The results showed that *CUL4B* was the direct target of miR-942-5p. When RA FLS was transfected with miR-942-5p mimics and inhibitors, miR-942-5p mimics significantly up-regulated the expression of miR-942-5p and inhibited the expression of *CUL4B*. On the contrary, after the transfer of miR-942-5p inhibitors, the expression of miR-942-5p decreased significantly (Figure 5D) and the expression of *CUL4B* increased (Figure 5E). Western blotting further confirmed that up-regulated miR-942-5p inhibited the *CUL4B* protein expression (Figure 5F), and knockout of miR-942-5p resulted in a significant increase in *CUL4B* expression (Figure 5G).

In order to further verify the regulatory relationship between miR-942-5p and *CUL4B*, *CUL4B* vectors were prepared. When CUL4B vectors were transferred into RA FLS, the expression of *CUL4B* mRNA (Figure 5H) and protein (Figure 5I) increased significantly. After transferring miR-942-5p mimics and *CUL4B* vectors into RA FLS, the cell proliferation rate was detected. The results showed that miR-942-5p mimics could significantly inhibit the cell proliferation at 24, 48 and 72 h after transfection, but *CUL4B* vectors could reverse the effect of miR-942-5p mimics (Figure 5J). Similarly, miR-942-5p mimics transfection significantly inhibited the expression of *β-catenin*, *C-myc* and *CCND1*, and *CUL4B* vectors reversed the effect of miR-942-5p mimics (Figure 5K). The above results further prove that *CUL4B* is the direct target of miR-942-5p, and miR-942-5p takes the *CUL4B* as the target to affect the canonical Wnt signal pathway in RA FLS.

### Effect of circ_0015756 on the miR-942-5p in Rheumatoid Arthritis Fibroblast-Like Synoviocytes

**FIGURE 6:**
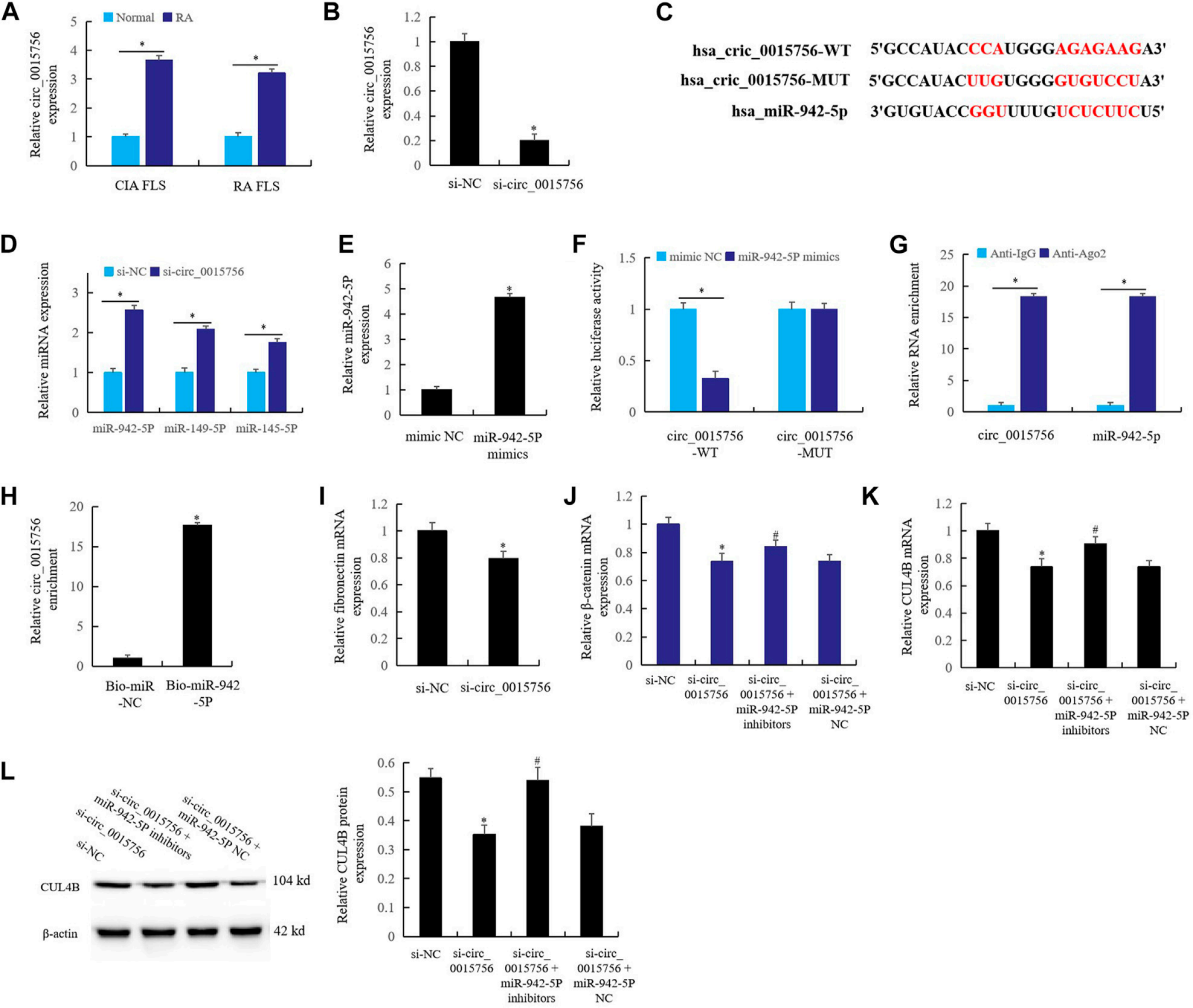
Effect of circ_0015756 on the miR-942-5p in RA FLS. **(A)** the expression of circ_0015756 in RA synovium and FLS; **(B)**: the expression of circ_0015756 decreased after knockout of circ_0015756; **(C)**: bioinformatics prediction; **(D)**: circ_0015756 knockout up-regulate the expression of miR-942-5p; **(E)**: the role of miR-942-5p mimics in the expression of miR-942-5p was detected by real-time qPCR; the direct interaction between circ_0015756 and miR-942-5p was detected by double luciferase reporter gene **(F)**, RIP assay **(G)** and RNA pull-down assay **(H)**; **(I)**: the effect of knockout of circ_0015756 on the *fibronectin* was detected by real-time qPCR; **(J–L)**: the effects of miR-942-5p silencing on the circ_0015756 knockout were detected by real-time qPCR and western blotting. For **(J–L)**, *Si-circ_0015756 group was compared with si-NC group; #si-circ_0015756+miR-942-5p inhibitor group was compared with si-circ_0015756 group.

In CIA FLS and RA FLS, the expression of circ_0015756 increased significantly (Figure 6A) compared with the normal group, and the expression of circ_0015756 decreased significantly after knockout of circ_0015756 (Figure 6B). Bioinformatics prediction (Figure 6C), combined with real time qPCR detection, found that circ_0015756 knockout could significantly up-regulate the expression of miR-942-5p (Figure 6D). This suggests that there may be a direct regulatory relationship between circ_0015756 and miR-942-5p.

When miR-942-5p mimics were added to RA FLS culture medium, the level of miR-942-5p increased significantly (Figure 6E). The direct interaction relationship between circ_0015756 and miR-942-5p was proved by double luciferase reporter gene (Figure 6F), RIP assay (Figure 6G) and RNA pull-down assay (Figure 6H). The results showed that the circ_0015756 had a direct regulatory effect on miR-942-5p.

Furthermore, knockout of circ_0015756 significantly reduced the expression of RA gene *fibronectin*, which confirmed that circ_0015756 could promote the pathogenesis of RA (Figure 6I). In RA FLS, knockout of the circ_0015756 inhibited the expression of *β-catenin* (Figure 6J) and *CUL4B* (Figures 6K,L). Meanwhile, knockout of circ_0015756 and miR-942-5p could reverse the effects of circ_0015756 knockout alone, which further confirmed the direct regulatory relationship of the circ_0015756 to miR-942-5p.

### The Interference Effects of Huangqin Qingre Chubi Capsule on the circ_0015756/miR-942-5p/*CUL4B*/*β-Catenin* Axis in Rheumatoid Arthritis Fibroblast-Like Synoviocytes

**FIGURE 7:**
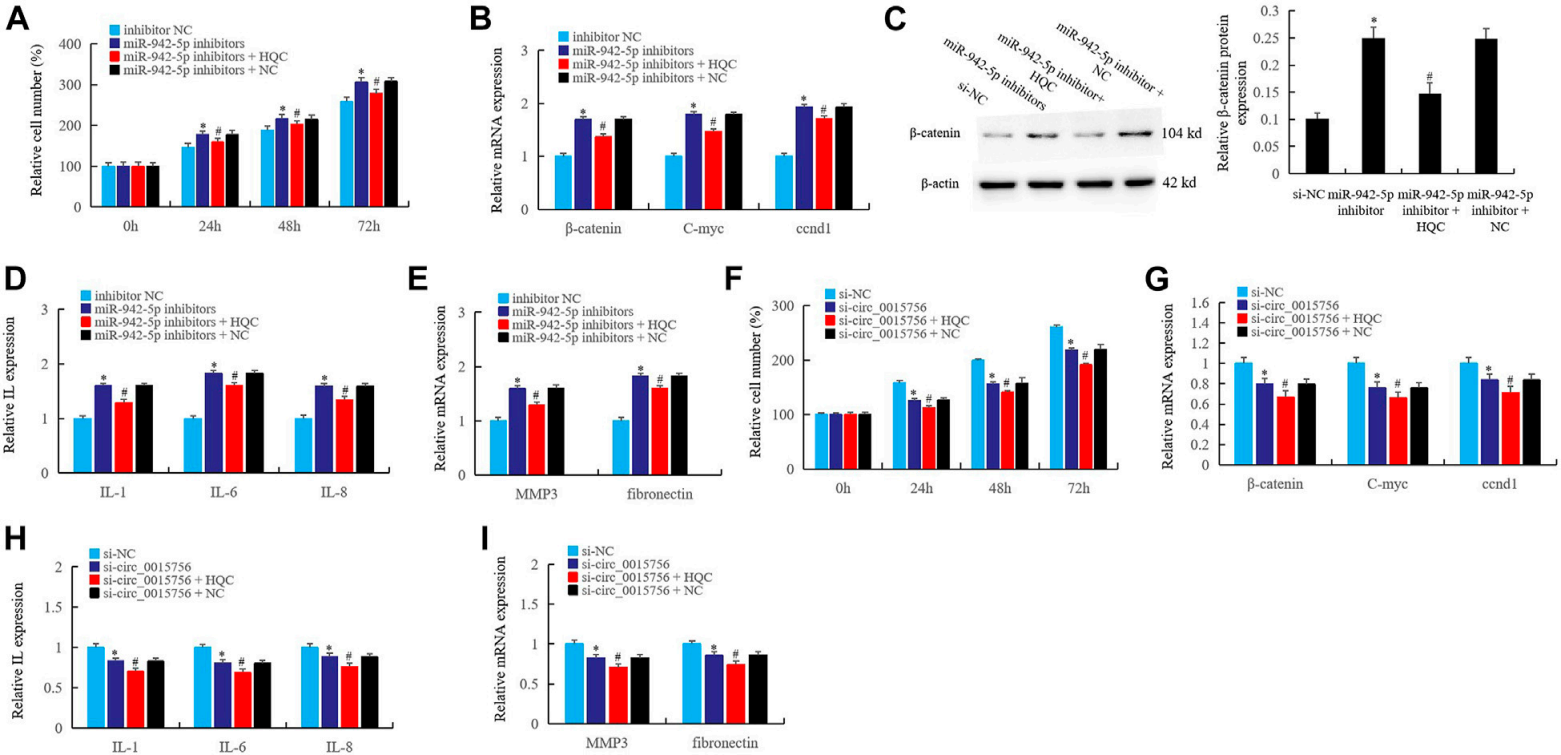
The interference effects of HQC on the circ_0015756/miR-942-5p/*CUL4B*/*β-catenin* axis in RA FLS. The interference effects of HQC on the circ_0015756/miR-942-5p/*CUL4B*/*β-catenin* axis was detected. **(A)**: cell counting; the interference of HQC on the miR-942-5p silencing was detected by real-time qPCR **(B)** and western blotting **(C)**; HQC interfered the miR-942-5p to affect the expression of IL-1, IL-6, IL-8 **(D)** and the *MMP3* and *fibronectin* **(E)** in RA FLS; **(F)**: HQC affected the cell proliferation activity of RA FLS by interfering the circ_0015756; HQC interfered the circ_0015756 and the influence on Wnt signal was detected by real-time qPCR **(G)**; HQC interfered the circ_0015756 to affect the expression of IL-1, IL-6, IL-8 **(H)** and the *MMP3* and *fibronectin* **(I)** in RA FLS. For **(A–E)**, *miR-942-5p inhibitor group was compared with inhibitor NC group; #miR-942-5p inhibitor + H QC group was compared with miR-942-5p inhibitor group. For **(F–I)**, *Si-circ_0015756 group was compared with si-NC group; #si-circ_0015756 + HQC group was compared with si-circ_0015756 group.

In the pathological mechanism of RA, circ_0015756 affects synovial proliferation and inflammation by targeting *CUL4B* through miR-942-5p, and HQC may take the *CUL4B* as the target to interfere with the circ_0015756. In this work, cell counting was used to detect the effects of HQC medicated serum (20%) on the miR-942-5p knockout, and the results showed that miR-942-5p knockout significantly enhanced the proliferation activity of RA FLS at 24, 48 and 72 h of miR-942-5p knockout, and HQC medicated serum (20%) could reverse the effects of miR-942-5p knockout (Figure 7A). Real time qPCR showed that the expression of *β-catenin, C-myc* and *CCND1* increased significantly after miR-942-5p knockout. HQC medicated serum (20%) could reverse the effects of miR-942-5p knockout on the expression of these factors (Figure 7B). The results of western blotting further confirmed the above findings (Figure 7C). After miR-942-5p knockout, the expressions of IL-1, IL-6, IL-8 (Figure 7D), and RA related genes *MMP3* and *fibronectin* (Figure 7E) were significantly increased. When HQC medicated serum (20%) was added, the levels of the above factors were significantly decreased. These results suggest that HQC can interfere with the roles of miR-942-5p in RA pathogenesis.

Further, after the circ_0015756 was knocked out, the proliferation activity of RA FLS decreased significantly at 24, 48 and 72 h, and the proliferation activity of FLS further decreased after HQC medicated serum (20%) was added (Figure 7F). When the circ_0015756 was knocked out, the expression of *β-catenin*, *C-myc* and *CCND1* was significantly inhibited. After HQC medicated serum (20%) was added, the expression of *β-catenin*, *C-myc* and *CCND1* was further inhibited (Figure 7G). HQC medicated serum (20%) can further enhance the inhibitory effects of circ_0015756 knockout on IL-1, IL-6, IL-8 (Figure 7H), *MMP3* and *fibronectin* (Figure 7I). This confirmed the effects of HQC targeting *CUL4B* on the circ_0015756/miR-942-5p/*CUL4B*/*β-catenin* axis, suggesting that HQC was an effective Traditional Chinese medicine compound for the treatment of RA.

## Discussion

RA is an autoimmune disease mainly characterized by chronic inflammation of synovium, which can cause joint swelling and pain, and then lead to narrowing of joint space, destruction of cartilage, joint deformity, and finally different degrees of disability (Maden, 2020; Salliot et al., 2020). According to statistics, RA has been worldwide incidence, the average incidence rate is 1%, the prevalence rate of China is 0.3–0.4%. If not diagnosed and treated in time, 70% patients will be disabled after 2 years, and the average life expectancy will be shortened by 10–15 years (Rooney et al., 2020; Deane and Holers, 2021). In order to prevent and slow down the destruction of joints and improve the long-term disease status of patients, anti rheumatic drugs are used to improve the disease tend to be used clinically, and the disease symptoms of patients have been better improved, but the condition of most patients can not be effectively controlled for a long time. The activation and proliferation of FLS in synovial lining layer is a central event in the occurrence of RA disease. Proliferative FLS releases the IL-6, IL-8, IL-15, fibronectin, MMP3, matrix degrading enzyme, etc., regulates the formation of pannus that destroys the cartilage, resulting in irreversible joint damage (McHugh, 2020a; van Delft and Huizinga, 2020).

FLS plays an important regulatory role in the pathogenesis of RA. The NFκB ligand RANKL expressed on FLS and T cells interacts with the common receptor RANK expressed on monocytes to initiate the differentiation of osteoclasts and bone resorption. Therefore, FLS can regulate the formation of pannus, resulting in irreversible joint damage (McHugh, 2020b). In addition, FLS has the proliferation characteristics of stem cells, and the abnormal synovial hyperplasia may result from the compensatory regeneration of FLS caused by joint injury. In view of the key roles of FLS in the pathogenesis of RA, FLS is used as the research object in the study of RA pathological mechanisms and RA drug therapy (Zhu et al., 2021).

We found that HQC improved the severity of arthritis in CIA mice. The indicators of improvement included the incidence rate, arthritis score, hind foot swelling and body weight. In the CIA mice we prepared, the incidence rate of the model group reached 100% in 40th days, and the incidence rate in the HQC treatment group reached 100% on the 52nd day. In the arthritis score and foot swelling score, from the 32nd day, the scores of the model group were significantly higher than those of the normal group, and the arthritis score and foot swelling score of the HQC group were significantly lower than those of the model group. Further, the pain sensitivity of CIA mice also decreased after HQC treatment. HE staining further verified that HQC had a good therapeutic effect on CIA mice.

HQC is a clinically effective traditional Chinese medicine for the treatment of RA invented and produced by the First Affiliated Hospital of Anhui University of Chinese medicine, which has a clear role in the treatment of RA (Guo et al., 2020a; Huang et al., 2020). Through our experimental study on CIA mice, it is confirmed that HQC has therapeutic effect on RA model mice. This also provides an animal model for us to study the molecular mechanisms of HQC in the treatment of RA.

The therapeutic effects of HQC on CIA mice in turn suggests that HQC may inhibit the pathological development of RA patients. In view of the core role of FLS in the study of pathological mechanisms of RA, we first studied the effects of HQC on the growth of FLS by cell counting and MTT. The results showed that compared with normal FLS, the proliferation of FLS in RA patients was significantly increased, and HQC could significantly inhibit the abnormal proliferation of FLS. ELISA showed that the levels of IL-1, IL-6 and IL-8 in RA FLS treated with HQC for 12, 24 and 36 h were significantly down-regulated. *MMP3* and *fibronectin* are pathology related genes of RA. The abnormal expression of these two genes indicates the development of RA pathology. We found that HQC significantly inhibited the expression of *MMP3* and *fibronectin* in RA FLS. These results further suggest that HQC has a therapeutic effect on RA.

Our previous research work proved that the expression of *CUL4B* in synovium and FSL of adjuvant arthritis rats (AA rats) was significantly increased, and was involved in the pathological mechanisms of RA (Miao et al., 2018ba). In this work, we detected the increase of *CUL4B* expression in CIA mouse synovium and FLS, and detected the effects of HQC on the *CUL4B* expression. The results showed that the expression of *CUL4B* in the synovium of CIA mice increased gradually with the extension of modeling time. Intragastric administration of HQC significantly inhibited the expression of *CUL4B* in the synovium of CIA mice. This suggests that HQC may inhibit the pathological development of RA by inhibiting the *CUL4B*.

HQC medicated serum (20%) was added to our cultured CIA FLS, and the expression of *CUL4B* increased gradually from the third generation to the sixth generation FLS. HQC drug serum (20%) significantly inhibited the expression of *CUL4B* in the pathogenesis of CIA mice. The results of immunohistochemistry and immunofluorescence showed that compared with the normal group, the expression of *CUL4B* in the synovium of CIA model mice increased significantly, and the expression of *CUL4B* decreased significantly after intragastric HQC. We found that from the third generation to the sixth generation of RA FLS, the expression of *CUL4B* in RA FLS gradually increased, and HQC significantly inhibited the expression of *CUL4B* in RA FLS. These findings suggest that HQC may affect the pathological development of RA by inhibiting the expression of *CUL4B*.

Wnt signal plays an important regulatory role in RA pathology. Wnt signaling pathway regulates and controls many life processes, including differentiation and maintenance of cell morphology and function, stress, immunity, cell carcinogenesis, apoptosis and anti apoptosis (Huang et al., 2019). More than a dozen known high-risk cancers stem from the imbalance of Wnt signal transduction pathway, and Wnt signal is also closely related to the occurrence of RA. The expression of β-catenin, a key gene of classical Wnt signaling pathway, was significantly increased in synovium of RA patients, suggesting that Wnt signaling is activated in the pathological process of RA (Rabelo et al., 2010). Wnt1, Wnt5a, wnt7b, and fz5 were significantly overexpressed in synovium of RA patients. The high expression of Wnt1, Wnt5a, and fz5 was also detected in joint FLS of RA patients. The highly expressed Wnt1, Wnt5a, and wnt7b bound to FZ receptors on cell membrane surface and regulated the pathological changes of RA through Wnt signal (Imai et al., 2009; Liu et al., 2019; Wang et al., 2020).

Our previous study found that DNA hypermethylation mechanism led to the decrease of *SFRPs* expression and the activation of Wnt signal. In the pathological mechanism of RA model rats, DNA methyltransferase 1 (DNMT1) mediates the hypermethylation of *SFRP4* and *SFRP2* promoter regions, and the enrichment and binding of methyl-CpG binding protein 2 (MeCP2) in the hypermethylation regions of *SFRP4* and *SFRP2* promoters, resulting in the inhibition of the expression of *SFRP4* and *SFRP2* and the activation of Wnt signaling pathway (Miao et al., 2015a; Miao et al., 2018b). CUL4B mediated mechanism affects the activity of Wnt signaling pathway. The abnormally high expression of CUL4B promotes the activation of Wnt signaling pathway, and there is a synergistic mechanism with PRC2 and EZH2. Meanwhile, miR-101-3p is the upstream regulator of *CUL4B*. The low expression of miR-101-3p directly leads to the abnormal high expression of *CUL4B*. Further, miRNAs mechanism affects the activity of Wnt signaling pathway. In RA model rats and RA patient samples, the expression of miR-152, miR-663, miR-375 and miR-148b-3p is abnormal. The abnormally expressed four miRNAs affect the Wnt signal pathway through their respective targets and further affect the expression of RA related genes (Miao et al., 2014; Miao et al., 2015b; Miao et al., 2015c; Miao et al., 2018c). Through recent studies, epigenetic modifications represented by DNA hypermethylation, miRNAs and CUL4B mediated ubiquitination can participate in the pathogenesis of RA by regulating the Wnt signaling pathway.

In this work, adding HQC medicated serum (20%) to the cultured RA FLS culture medium could inhibit the expression of *β-catenin*, and the expression of *c-Myc* and *CCND1* also decreased significantly, indicating that HQC inhibits the canonical Wnt signaling pathway in the pathogenesis of RA. *CUL4B* vectors reversed the effects of HQC medicated serum (20%), suggesting that *CUL4B* played a key role in the mechanisms of HQC in the treatment of RA, and HQC may play its role through the *CUL4B*.

Through bioinformatics prediction and experimental verification, in the pathological mechanisms of RA, circ_0015756 targeted the *CUL4B* through the miR-942-5p to affect the synovial proliferation and inflammation of RA. In view of the regulatory effects of HQC on the *CUL4B*, HQC may target the *CUL4B* and interfere with the effect of circ_0015756. Studies have shown that HQC medicated serum (20%) could reverse the effects of miR-942-5p gene knockout. After miR-942-5p knockout, the expressions of *β-catenin, c-Myc* and *CCND1* increased significantly. HQC medicated serum (20%) could reverse the effects of miR-942-5p knockout on the expression of these factors. Further, when HQC medicated serum was added, the levels of L-1, IL-6 and IL-8 decreased significantly. These results suggest that HQC can interfere with the roles of miR-942-5p in the pathogenesis of RA.

When circ_0015756 was knocked out, the expressions of *β-catenin, c-Myc* and *CCND1* were significantly inhibited. After adding HQC medicated serum, the expressions of *β-catenin, c-Myc* and *CCND1* was further inhibited. HQC medicated serum could further enhance the inhibitory effects of circ_0015756 gene knockout on IL-1, IL-6, IL-8, *MMP3* and *fibronectin*. This confirmed the effects of HQC on the circ_0015756/miR-942-5p/*CUL4B*/*β-catenin* axis by targeting the *CUL4B*, indicating that HQC is an effective traditional Chinese medicine compound for the treatment of RA.

## Data Availability

The data analyzed in this study is subject to the following licenses/restrictions: Some data can be provided after receiving the editing request. Requests to access these datasets should be directed to CM, miaocg@ahtcm.edu.cn.

## Abbreviations

AA rats, adjuvant arthritis rats; CRL, CUL4 subfamily of cullin-really interesting new gene (RING) ubiquitin ligase; CUL4B, Cullin 4B; DNMT1, DNA methyltransferase 1; DMSO, dimethyl sulfoxide; FBS, fetal bovine serum; FLS, fibroblast-like synoviocytes; HQC, Huangqin Qingre Chubi Capsule; MeCP2, methyl-CpG binding protein 2; MTX, Methotrexate; MTT, 3-(4,5-dimethylthiazol-2-yl)-2,5-diphenyltetrazoliumbromide; RA, rheumatoid arthritis; RIP, RNA immunoprecipitation; SDF-1, stromal cell-derived molecule.
